# Supplementary material for: Monitoring Apricot (Prunus armeniaca L.) Ripening Progression through Candidate Gene Expression Analysis
Source: Int J Mol Sci. 2022 Apr 20;23(9):4575. doi: 10.3390/ijms23094575 (PMC9105867; doi:10.3390/ijms23094575)
Supplement: Supplementary file 1 [file ijms-23-04575-s001.zip › ijms-1678625-supplementary.pdf]

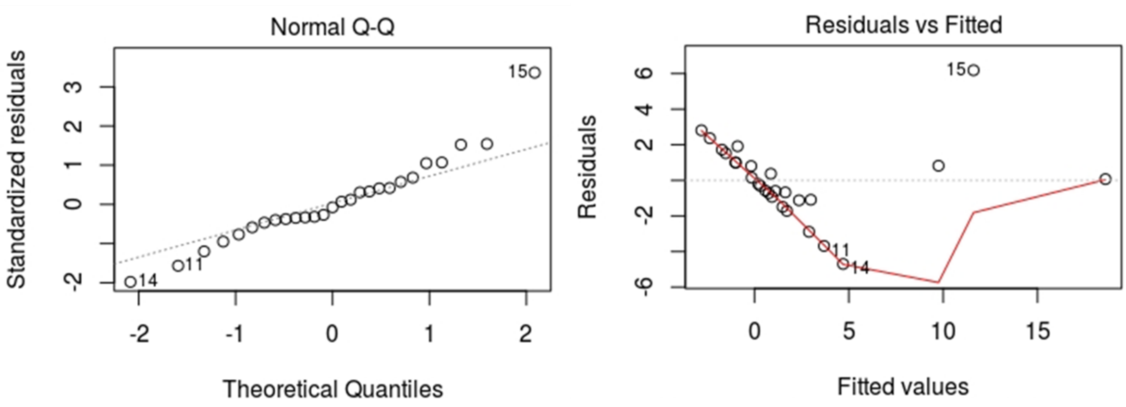

Figure S1. Diagnostic plots of MLR analysis for anthocyanins content. Q-Q plot (left) and residuals vs fitted values plot (right).

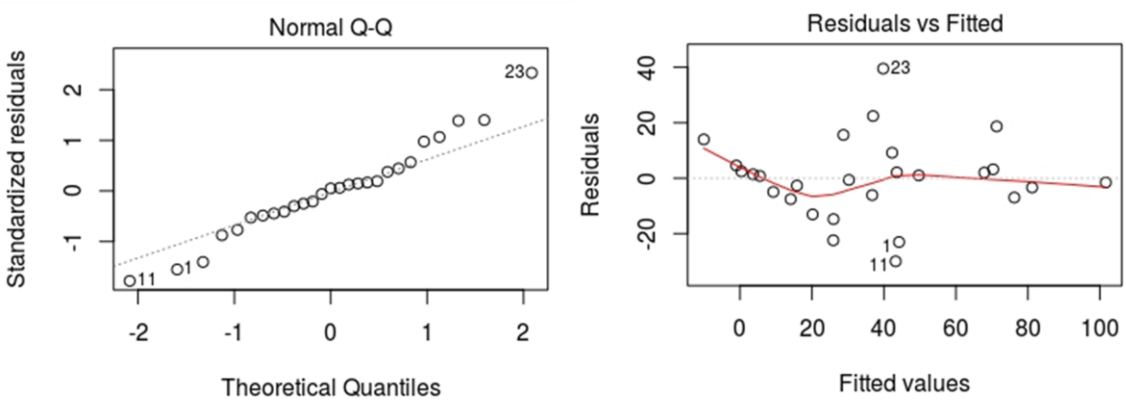

Figure S2. Diagnostic plots for MLR analysis for carotenoids content. Normal Q-Q plot (left) and residuals vs fitted values plot (right).

Table S1. qRT- Table S1. PCR primers for candidate genes related to phenological and quality traits during the ripening process in apricot fruit and housekeeping genes. Ensemble identification of closer gene is referred to peach (*Prunus persica* (L.) Basch.) reference genome [*Prunus persica* genome v2.0.a1] and Japanese apricot (*Prunus mume* Siebold & Zucc.) reference genome [*Prunus mume* genome v1.0].

Name	***P.persica*** **Ensembl gene id.**	***P.mume*** **Ensembl gene id.**	**PCR product location**	**Forward**	**Reverse**	**Annealing temperature (°C)**	**Description**
18 S RNA SUBUNIT (S18)	NA	NA	NA	GTTACTTTTAGGACTCCGCC	TTCCTTTAAGTTTCAGCCTTG	54.3	Housekeeping gene
1-DEOXY-D-XYLULOSE-5-PHOSPHATE SYNTHASE (DXS)	Prupe.6G204700	LOC103335117	Pp06:21280553..21280665	ACCTTGAGTGGTCCATCTAGAAT	GCCAAGGAGCATGAAATCCT	59.02	Isoprenoids biosynthesis.
4-COUMARATE COA LIGASE (4CL)	Prupe.3G107300	LOC103328252	Pp03:8556093..8556206	GATGGGGAGAATCCCAACTT	CCAGCTCTTAGCCCACAAAG	63.8	Anthocyanin biosynthesis
UDP FLAVONOID 3-O-GLUCOSYLTRANSFERASE (UFGT)	Prupe.6G190100	LOC103323450	Pp06:19754156..19754308	TCAGGCCTAACAATCCACAA	GAATGTTTGAAATGGCTTGA	59.12	Anthocyanins biosynthesis
ANTHOCYANIDIN SYNTHESE (ANS)	Prupe.5G086700	LOC103337372	Pp05:9817305..9817427	AGGAGTTGAAGAAGGCAGCA	GCTCAATGGGAAGATCGAAA	55.3	Anthocyanin biosynthesis
BASIC HELIX-LOOP-HELIX DNA-BINDING DOMAIN (bHLH)	Prupe.6G343400	LOC103318640	Pp06:29556398..NA	CCCAGCTTTAATTCCTTGTC	CACAGCCAAGATCTGCTCAA	59.95	Trancription factor

Table S1. Continuation.

Name	*P.persica* Ensembl gene id.	*P.mume* Ensembl gene id.	PCR product location	Forward	Reverse	Annealing temperature (°C)	Description
CAPSANTHIN/CAPSORUBIN SYNTHASE, CHROMOPLASTIC-LIKE (CrtL)	Prupe.6G135100	LOC103328471	Pp06:10748411..10748529	CGTGATCATCATCGGCACTG	TGGCCACAAAGATTGCGGTG	60.64	Carotenoid biosynthesis
CAROTENOID CLEAVAGE DIOXYGENASE 1 (CCD1)	Prupe.2G014700	LOC103330446	Pp02:NA..1307443	AACAGCTGGCTTACTGGATTATGA	GTAAACATCTCGCCAGTGAATGG	59.3	Carotenoid degradation.
CAROTENOID CLEAVAGE DIOXYGENASE 4 (CCD4)	Prupe.1G255500	LOC103321735	Pp01:26615571.26615664.	TCGGTTGAGAAAGTTAGGATTGATC	CGGATTGAACACAGCAAAGTCTA	58.9	Carotenoid degradation.
CHALCONE/STILBENE SYNTHASE (CHS)	Prupe.I005800	LOC10332431	scaffold_71:9583..9721	CCGTGAAGTTGGGCTTACAT	TGGGTGTGCAATCCAGAATA	55.3	Carotenoid biosynthesis
TRANS-CINNAMATE 4-MONOOXYGENASE (C4H)	Prupe.6G040400	LOC103339178	Pp06:3019257..3019356	GAAGCTCAAGGGGTTGAATG	GCCTCTCAAGAAGGGTCTCA	63.5	Anthocyanin biosynthesis
CINNAMYL-ALCOHOL DEHYDROGENASE (CAD1)	Prupe.6G208200	LOC103335323	Pp06:21675954..21676090	CTTGGTAGCAGCCAAGGTGT	CCGCAAAACACAACATAACG	60.03	Phenylpropanoids biosynthesis.

Table S1. Continuation.

Name	*P.persica* Ensembl gene id.	*P.mume* Ensembl gene id.	PCR product location	Forward	Reverse	Annealing temperature (°C)	Description
COMMON PLANT REGULATORY FACTOR 1-LIKE (bZIP)	Prupe.2G182800	LOC103332281	Pp02:22520584..22520605	GCGTAGGACGAATGGAAGAG	ATCCCTAGCAATGGAACCCG	59.15	Transcription factor
CONSERVED WD40 REPEAT-CONTAINING PROTEIN (WD40)	Prupe.6G161500	LOC103338045	Pp06:15025150..15025276	TGCTGAAATGCTGCCTTGTA	CAATTACAGTGTGCCCAAATTC	59.38	Transcription factor
CYCLOPHILIN (CYP1)	Prupe.8G161100	LOC103334995	Pp08:16951241..16951361	CAACGGATCTCAGTTCTTCGTCTGC	GACCCAACCTTCTCGATGTTCTTCA	59.4	Housekeeping gene
DIHYDROFLAVONOL 4-REDUCTASE (DFR)	Prupe.1G310300	LOC103320869	Pp01:30170232..30170313	GTGGTCCGGACAAGATGACC	GTGTAACTGGAGGAACAGGT	59.63	Flavonoid biosynthesis
FLAVONOID 3'5'-HYDROXYLASE 1-LIKE (F3'5'H)	Prupe.5G078100	LOC103337305	Pp05:9179755..9179904	ACTATTTACCATTCGGGTCA	CAAACTTCTCTGAAAGATCC	58.88	Anthocyanin biosynthesis
GLUTATHIONE TRANSFERASE GST 23-LIKE (GST)	Prupe.1G054800	LOC103323820	Pp01:3863891..3863993	TCACACCCTTGAGCTTTAGA	TCTCAACACGTCAACAATGG	60.14	Detoxification and antioxidant related protein
LIPOXYGENASE 2 (LOX2)	Prupe.4G047800	LOC103324771	Pp04:2285617..2285711	CTGGTACAACGAGTCAATCAATTTAGG	AATGGATGTAAGATCATCTGGAGTAGAG	64.4	Esters biosynthesis (VOCs)

Table S1. Continuation.

Name	*P.persica* Ensembl gene id.	*P.mume* Ensembl gene id.	PCR product location	Forward	Reverse	Annealing temperature (°C)	Description
MADS BOX TRANSCRIPTION FACTOR (MADS BOX)	Prupe.1G549600	LOC103331940	Pp01:44926224..44926314	CTTCAGCAGTCCATTTCTTCG	GATGGGGAGAGGAAAGATTGA	60.00	Transcription factor
MULTIDRUG AND TOXIN EFFLUX TRANSPORTERS (MATE)	Prupe.1G320200	LOC103333172	Pp01:NA..30712879	GGCGAAAACGATTGAGCTTA	TGACGAATCTTCTTAGGTTTGTG	58.44	Membrane transporter
MYB TRANCRIPTION FACTOR (MYB10)	Prupe.3G163000	NA	Pp03:18183819..18184015	GGCAGGACTGCGAATAATGT	CCCACCAATCACGTTGAGTA	57.3	Anthocyanin biosynthesis
NAC DOMAIN-CONTAINING PROTEIN 72-LIKE (NAC)	Prupe.4G186800	LOC103326246	Pp04:11118077..11118268	AGAACTCAGCGGGTTGATAACT	TGCACCCCTACTCGATTTCT	57.3	Transcription factor
PROBABLE SERINE/THREONINE-PROTEIN KINASE PBL16 (WRKY)	Prupe.6G002400	LOC103337200	Pp06:301784..301884	GCCATTCTCTGTGTCCTTGA	GGGTTCATCACTGAGGATTT	58.97	Transcription factor.
PSBP DOMAIN CONTAINING PROTEIN 6, CHLOROPLASTIC (psbP1)	Prupe.4G213200	LOC103326460	Pp04:13294576..13294680	GCTTCCTCTTGCTCTGGTTG	AAGGTGAGGGTGGGAGGTAG	60.13	Photosynthesis
RIBOSOMAL PROTEIN L12 (RPL12)	Prupe.6G101700	LOC103325279	Pp06:NA..7075090	CGATCCCTCACAGGTCGTCG	TCCAGTCGTTGGCGGTCTCC	62.32	Housekeeping gene

Table S1. Continuation.

Name	*P.persica* Ensembl gene id.	*P.mume* Ensembl gene id.	PCR product location	Forward	Reverse	Annealing temperature (°C)	Description
SERINE/THREONINE-PROTEIN KINASE EDR1-LIKE (EDR1)	Prupe.3G117600	LOC103328501	Pp03:10062203..10062356	GGACAGAACCGGATGATCGG	CAGAATACAACCTTAAGCCGAGA	59.79	Growth regulator (ethylene)
ZETA CAROTENE DESATURASE (ZDS)	Prupe.6G340000	LOC103318575	Pp06:29399216..29399583	GGACAAGGTTCACTCCTCCA	TGGGATGATGGGAATAAAGC	63.5	Carotenoid biosynthesis
